# A Case of Death from Chloral Hydrate

**Published:** 1871-02

**Authors:** William Holbrook

**Affiliations:** Palmer, Mass.


ART. VII.—*A Case of Death from Chloral Hydrate.* By WILLIAM HOLBROOK, M. D., of Palmer, Mass.

Mrs. D——, aged twenty-four, the mother of two children, of which the younger is but four month's old, took a dose of chloral hydrate, at about nine o'clock on the evening of February 10th. I was called to see her at half-past nine the next morning, and found her breathing stertoriously. Her pupils were contracted, and insensible to the light: the conjunctiva was congested. Her countenance was livid, and her extremities were cold. She was lying on her right side, and no pulse was to be detected at the wrist of that side; and that felt at the left wrist was small and thready. The heart's action was very feeble.

For treatment, I gave twenty drops of aromatic spirits of ammonia, repeated every fifteen minutes; and had mustard applied to the stomach, the whole length of the spine, and to both legs. Bottles of hot water were also placed to her sides, thighs, under both knees, and to her feet. The patient died at quarter past twelve at noon, having been wholly unconscious all the time. The heart's action increased in strength, and the pulse in volume, for an hour, during the treatment, then grew feeble till death occurred. It was with great difficulty that she could at first swallow, and frothy mucus was flowing from her mouth.

The lady was a wife of a reverend gentlemen of this place. Her husband had used only three doses from an ounce bottle of chloral, and she deliberately dissolved and took the balance of the drug herself. Her husband was away for the night, at the time when she took the fatal dose. She gave directions to the young woman who slept with her to take care of the babe and not to wake her.

Was it a case of suicide? How could any one take such a heroic dose of this chloral, while thirty grains is as much as I am able to get one to take, and that with much difficulty. As near as we could estimate, she took over four hundred grains of chloral in half a goblet of water.

No *post mortem* was made. What pathological conditions would have presented, had an examination been made? Vital functions must have ceased first in the brain, then in the circulation, and then in the heart and respiration. What treatment is of any avail in such cases, and what means are most efficient?

